# Two‐Way Referrals in Chinese Medical Consortia Under the Framework of Hierarchical Diagnosis and Treatment: Progress and Challenges

**DOI:** 10.1111/jebm.70069

**Published:** 2025-10-10

**Authors:** Xinyu Cui, Kexin Liu, Yuanyi Ji, Su Han, Yongzhong Cheng

**Affiliations:** ^1^ West China School of Public Health and West China Fourth Hospital Sichuan University Chengdu China; ^2^ West China Hospital Sichuan University Chengdu Sichuan China

**Keywords:** China, effect evaluation, medical consortium, two‐way referral

## Abstract

The hierarchical diagnosis and treatment system's two‐way referral mechanism is crucial for optimizing medical resource allocation, with medical consortia significantly enhancing this process. It evaluates the implementation dynamics of two‐way referral systems within China's medical consortia from 2019 to 2024, focusing on policy evolution, regional disparities, and stakeholder engagement. Despite a positive overall trend, referral rates remain low, with notable regional and institutional disparities. The implementation outcomes span various dimensions, including referral metrics, specific services, timing, and costs; however, these effects are inconsistent and warrant further investigation. The current evaluation index system is diverse but tends to prioritize quantity over quality. Additionally, awareness and satisfaction levels among medical personnel and patients regarding the two‐way referral system are uneven and influenced by multiple factors. Currently, China's two‐way referral system faces challenges, including low referral volumes, an inadequate evaluation framework, limited research perspectives, and insufficient motivation for stakeholder participation. Future efforts should focus on strengthening primary care infrastructure, enhancing resource collaboration, advancing health insurance reforms, refining the evaluation system, and fostering synergy between hierarchical diagnosis and treatment, the Healthy China strategy, and referral model innovation to advance the hierarchical diagnosis and treatment system. Recommendations emphasize strengthening primary care capacity, reforming insurance payment models, and leveraging digital health technologies to align with the “Healthy China 2030” strategic goals.

## 1 Introduction

Globally, most nations implement a hierarchical healthcare system, with many adopting this structure to ensure efficient and orderly access to medical services [1]. Countries such as the United States and the United Kingdom have established a rational three‐tier referral model comprising community health centers (family doctors), general hospitals, and large‐scale medical centers, each with distinct responsibilities [2, 3]. Similarly, China's healthcare system is organized into three levels [4]. However, the absence of a mandatory or systematic referral process results in an inverted triangle of health resource allocation and fragmentation in healthcare delivery. Patients often bypass lower‐tier facilities in favor of large medical centers, undermining the continuity of care essential for managing chronic noncommunicable diseases [5]. Since its formal integration into China's healthcare reform agenda in 2009, the hierarchical diagnosis and treatment (HDT) system has been positioned as a cornerstone for addressing resource fragmentation and optimizing service delivery [6]. The two‐way referral mechanism has been a crucial component in achieving hierarchical diagnosis and treatment. By forming healthcare consortia that integrate resources across various levels of healthcare institutions, this system facilitates the rational allocation of patients among these institutions [7]. Recently, the Chinese government has implemented policies to bolster the development of healthcare consortia and the two‐way referral system, thereby advancing these practices [8]. In 2023, the “Opinions on Further Improving the Healthcare Service System” was released, advocating for more precise objectives, systematic continuity in service models, and the development of a high‐quality, efficient healthcare system with Chinese characteristics [9]. While international models (e.g., the UK's National Health Service and the US Accountable Care Organisations) emphasize mandatory referrals and standardized pathways, China's HDT system remains predominantly policy‐driven, with voluntary participation and significant regional heterogeneity. This divergence highlights the importance of examining China's distinct institutional context to inform global health policy discussions. This paper examines the current status, impact, and evaluation of two‐way referrals within China's medical consortia over the past five years. The goal is to assess the state of these referrals, offer recommendations for advancing hierarchical diagnosis and treatment, integrate healthcare systems in China, and provide insights for similar efforts in other countries and regions.

## 2 Analysis of the Current Situation of Two‐Way Referrals in China

### 2.1 Development Overview

Since 2009, China has aimed to establish a coordinated division of labor and enhance its medical and healthcare service system by exploring a hierarchical diagnosis and treatment model with two‐way referrals tailored to national conditions [10]. A series of policies has been enacted to facilitate the reallocation of service resources, emphasizing “primary diagnosis and treatment at the grassroots level, two‐way referrals, separation of emergencies and chronic diseases, and integrated linkages” [11]. The evolution of government policies can be categorized into three phases. The first phase, from 2000 to 2009, was exploratory, during which certain regions piloted the formation of hospital clusters to address the uneven distribution of healthcare resources and the limited capacity of primary healthcare services. These initial consortia were primarily informal collaborations, with referrals and consultations conducted through alliance agreements, lacking a unified management and coordination framework. Between 2010 and 2017, during the policy promotion phase, the state prioritized the development of healthcare consortia. This period included the introduction of numerous policies aimed at deepening healthcare system reforms, thereby providing robust support for healthcare consortia and establishing a foundation for bidirectional referrals. Notably, in 2017, the General Office of the State Council issued a guiding opinion on advancing the construction and development of medical consortia, signaling the transition of bidirectional referrals into a phase of standardization and institutionalization [12]. Since 2018, the comprehensive promotion phase has been underway. The 2020 release of the Notice on the Issuance of Management Measures for Medical Consortia (for Trial Implementation) consolidated the experiences from pilot projects across regions, accelerating the development of medical consortia [13]. This initiative has facilitated the grid layout and management of these consortia, established a robust policy framework, and expedited two‐way referrals within China's hierarchical diagnosis and treatment system. Notably, in the past five years, supportive policies related to telemedicine, medical security, medical quality, and drug security have been introduced, ensuring effective labor division, service optimization, hierarchical diagnosis and treatment promotion, and system integration advancement.

China's graded diagnosis and treatment, along with its two‐way referral system, have advanced in terms of policy framework development and local pilot implementation but remain in the “hurdle‐climbing” phase overall. Future efforts must focus on enhancing grassroots capacity, overcoming interest barriers, and refining coordination mechanisms to transition from a “policy‐driven” to a “spontaneous mechanism” (Table 1).

**TABLE 1:** Development of hierarchical diagnosis and two‐way referral policies in China in recent years.

No.	Year	Policy name	Thrust
1	2009	Opinion on Deepening the Reform of the Medical and Health Service System	Proposed the establishment of a collaborative mechanism to improve the new medical and health service system
2	2010	Guiding Opinions on Pilot Reform of Public Hospitals	Exploring the implementation of hierarchical medical care and two‐way referrals
3	2015	Outline of the National Healthcare Service System Plan (2015–2020)	Clarifying the functional positioning of medical institutions at all levels, establishing a model of hierarchical diagnosis and treatment, improving the mechanism for the division of work and coordination among medical institutions, and realizing primary care at the grassroots level and two‐way referrals, among other things
4	2015	Guiding Opinions on Pilot Comprehensive Reform of Urban Public Hospitals	Exploring the division of labor and collaboration model for building medical consortia and their accompanying medical insurance policy
5	2016	Outline of the “Healthy China 2030” Plan	Implementing the construction of medical consortia, building a collaborative mechanism for medical and healthcare organizations, establishing a mature system of hierarchical diagnosis and treatment, and forming a reasonable order for medical treatment
6	2016	Guiding Opinions on Carrying Out Pilot Work on Medical Consortium Construction	Establishing the general requirements and basic principles for the construction of medical consortia, and gradually forming a scientific organizational model for medical consortia
7	2017	Guiding Opinions on Promoting the Construction and Development of Medical Consortia	Launching various forms of pilot construction of medical consortia
8	2017	Guiding Opinions on Further Deepening the Reform of Basic Medical Insurance Payment Methods	Combined with the construction of the hierarchical diagnosis and treatment model and the family doctor contracting service system, it guides the insured to prioritize the first consultation at the grassroots level. It explores the total payment of medical insurance
9	2017	On the issuance of the Action Plan for Further Improving Medical Services (2018–2020)	Strengthening medical quality control in all aspects of continuous medical services in the medical association
10	2018	Opinions on Reforming and Improving the Incentive Mechanism for the Training and Use of General Practitioners	Improving the system of training general practitioners that adapts to the characteristics of the profession
11	2018		
	Opinions on Promoting the Development of “Internet+Medical Health”	Encourages Medical Consortia to Actively Utilize Internet Technology	
12	2018	Notice on the Work Program for Comprehensive Performance Assessment of Medical Consortia (for Trial Implementation)	Harmonize performance assessment standards, assessment processes and assessment requirements, and objectively and scientifically assess the effectiveness of medical consortium construction
13	2018	Notice on Further Improving the Key Work Related to the Construction of the Graded Diagnosis and Treatment System	It further clarifies the requirements for the key work on the construction of the hierarchical diagnosis and treatment system, and clarifies the work objectives and tasks for the coming period
14	2019	Notice on Promoting the Construction of Close‐Knit County Medical and Healthcare Communities	To further improve the county medical and healthcare service system, improve the allocation and utilization efficiency of county medical and healthcare resources, and accelerate the enhancement of primary medical and healthcare service capacity
15	2019	Circular on Carrying Out Pilot Work on the Construction of Urban Medical Consortia	Gradually realize the grid layout management of urban medical consortiums and carry out pilot work on the construction of urban medical consortiums
16	2020	Circular on the Issuance of Measures for the Management of Medical Consortia (for Trial Implementation)	Fully summarize the experience of construction pilot work in various places: accelerate the construction of medical consortiums and gradually realize the grid layout and management of medical consortiums
17	2020	Notice on Issuance of Judging Criteria and Monitoring Indicator System for the Construction of Close‐knit County Medical and Healthcare Communities (for Trial Implementation)	Focusing on the key areas and key links of county medical community construction, regularly monitoring the progress and effectiveness of county medical community construction around the world, and further enhancing the capacity of county and grassroots medical and healthcare services
18	2021	Opinions on Promoting the High‐Quality Development of Public Hospitals	Constructing a new system for the high‐quality development of public hospitals and playing the leading role of public hospitals in the medical association
19	2021	Notice on Promoting Sanming City's Experience in Graded Diagnosis and Treatment and Medical Consortium Construction	Give full play to the role of typical demonstration and guidance for localities to learn from Sanming's experience and accelerate the improvement of the hierarchical diagnosis and treatment system according to local conditions
20	2022	Administrative Measures for Mutual Recognition of Examination and Test Results among Medical Institutions	Promoting the realization of mutual recognition of examination and test results among different medical institutions
21	2023	Opinions on Further Improving the Healthcare Service System	Strengthening division of labor, promoting hierarchical diagnosis and treatment, and promoting system integration
22	2024	Circular on Strengthening the First Consultation and Referral Services and Enhancing the Continuity of Medical Services	Promoting the construction of a hierarchical diagnosis and treatment system, further strengthening the first‐visit and referral services, enhancing the continuity of medical services, and improving the patients' experience of medical treatment
23	2024	Guiding Opinions on Further Promoting Mutual Recognition of Examination and Test Results in Medical Institutions	Strengthen system design, clarify the scope of mutual recognition, strengthen technical support, enhance the effectiveness of mutual recognition work, and standardize the behavior of medical examination and testing.

*Note*: All policy documents are from the State Council Policy Documents Library on the Chinese government website: https://www.gov.cn/zhengce/zhengcewenjianku/.

By the end of 2023, China had established over 18,000 medical consortia in various forms [14]. In 2024, nationwide two‐way referrals totaled 36,567,000, marking a 20.6% increase from 2023. Referrals to higher‐level hospitals decreased by 3.1% to 15,111,000, whereas referrals from higher‐level to lower‐level institutions rose by 45.7% to 21,456,000. This indicates a positive trend in the two‐way referral system, facilitating the vertical flow of medical resources and patient referrals and thereby creating a new healthcare model. The decline in upward referrals and the significant rise in downward referrals suggest improvements in the service level and capacity of primary medical institutions. Nonetheless, due to constraints such as limited grassroots capacity, benefit distribution imbalances, and patient mistrust, the two‐way referral rate remains below 5%, which is low compared to the 10%‐30% rates reported in other developed and developing countries [15] (Table 2).

**TABLE 2:** China national trends in two‐way referral volumes (2022–2024).

Year	Number of two‐way referrals (10,000)	Increase/decrease (%)	Number of upward referrals (10,000)	Increase/decrease (%)	Number of downward referrals (10,000)	Increase/decrease (%)
2022	2984.70	19.3^*^	1631.77	4.5^*^	1133.33	56.2^*^
2023	3032.17	9.7%	1559.97	−4.4	1472.20	29.9
2024	3656.70	20.6%	1511.10	−3.1	2145.60	45.7

* Compared with the end of China's 13th Five‐Year Plan for National Economic and Social Development (2016–2020).

Data sourced from public information.

Upward referrals: primary → tertiary; Downward referrals: tertiary → primary.

### 2.2 Significant Interregional and Interagency Differences

Despite the overall positive trend in the two‐way referral system of medical consortia, significant disparities and inadequacies persist across regions and institutions [16]. Sanming city, a model for promoting best practices, experienced a nearly 37‐fold increase in downward referrals and a nearly five‐fold increase in upward referrals from 2016 to 2019 [17]. Between 2014 and 2016, referrals from the 26 member units of the Peking University Third Hospital Medical Triad to its core unit rose annually, with upward referrals increasing by 106.5%, totaling 2507 cases [18]. In Bozhou City, Anhui Province, monitoring data from 2015 to 2020 indicated that the downward referral rate rose from 0.08% to 2.25%, while the upward referral rate increased from 0.72% to 7.66%. During this period, 21 medical consortia were established, encompassing 104 medical institutions, thereby enhancing resource allocation under a hierarchical diagnosis and treatment model [19]. Shanxi Province developed a medical network centered around approximately 10 core and tertiary hospitals, serving approximately 30 million residents, which facilitated the referral of complex cases from grassroots hospitals to advanced hospitals and alleviated patients' economic burdens [20].

In economically developed regions, resident participation in two‐way referrals is greater, with correspondingly high satisfaction levels. Conversely, in less developed areas, the effectiveness of two‐way referrals is constrained by limited informatization and inadequate grassroots service capacity. This study revealed a significant positive correlation between regional economic development and referral effectiveness [21]. For example [22, 23], regions such as the Yangtze River Delta and the Pearl River Delta have enhanced referral quality and effectiveness through institutional innovation. Medical consortia have enhanced referral efficiency by integrating resources, optimizing service processes, and leveraging innovations in information technology and health insurance payment, such as composite payment models under total control. Through economic‐benefit coordination, hospitals and residents are encouraged to engage in hierarchical diagnosis and treatment [24]. The Shenzhen Luohu two‐way referral system in Guangdong Province significantly facilitates the integration of medical resources and information sharing within the region. Concurrently, the implementation of a hierarchical management system for medical expenses and a differential payment system for medical insurance—such as requiring second‐ and third‐tier insurance participants to initiate consultations at community health service centers—has effectively enhanced primary medical service capacity. A study involving 1436 breast cancer patients from 26 county‐level hospitals in China revealed that 1172 (81.62%) were initially diagnosed at these hospitals, with 81 (6.91%) subsequently referred to higher‐level facilities. Most patients (75.99%, 1092/1436) completed the diagnostic and treatment process at county hospitals, indicating that enhanced grassroots capacity enables these hospitals to independently manage the entire diagnostic and treatment process [25]. In certain remote regions [15], the effectiveness of two‐way referrals is constrained by insufficient medical resources, limited medical capacity, and inadequate information technology. This is particularly evident in economically underdeveloped county‐level medical consortia, where doctors' referral practices are shaped by subjective norms and perceived behavioral control; however, overall referral activity remains infrequent [26].

Internet healthcare has significantly facilitated two‐way referrals, enhancing referral efficiency through online outpatient clinics, remote consultations, and e‐prescription sharing, although the overall business volume remains low. By building a referral platform, Ningbo City, Zhejiang Province, has achieved the targeted opening of specialist numbers for grassroots appointments, which account for 20% of the total number of appointments. From 2015 to 2017, the number of outpatient visits from lower‐level hospitals increased from 280 and 1236 to 1950; at the same time, the number of inpatients referred from lower‐level hospitals increased from 51 and 300 to 712 [27]. In Beijing, the referral system construction rate is notably higher in tertiary hospitals (38.3%) than in secondary hospitals (28.6%), highlighting the entrenched hierarchy in medical resource allocation [28]. In the field of maternity and child healthcare, a study on IT‐based two‐way referrals in Minhang District, Shanghai, revealed that the intervention group achieved superior outcomes in the early diagnosis and management of cervical lesions compared with the control group [29]. A survey in Anhui Province revealed that only 22.08% of 77 maternity and child healthcare institutions had implemented telemedicine systems, primarily for remote consultations and imaging diagnoses, indicating the underutilization of telemedicine's full potential. Two‐way referral information platform coverage remains limited, and an effective referral network has yet to be established. The frequency of upward referrals for outpatient and inpatient services is three times greater than that of downward referrals, with grassroots maternal and child healthcare institutions favoring upward referrals [30]. In Hubei Province, a study revealed that most maternity services flow to secondary and tertiary hospitals within municipal units in the western region. In contrast, many mothers in the central‐eastern area travel across towns for care [31].

## 3 Effectiveness of the Implementation of Two‐Way Referrals by Medical Consortia

The effectiveness of implementing two‐way referrals in healthcare consortia requires comprehensive, multidimensional indicators that cover not only efficiency indicators, such as referral volume and referral rate, but also various aspects, including resource utilization, economic operation, quality, safety, and patient experience.

### 3.1 Operational and Economic Related Indicators

Several studies have demonstrated the beneficial effects of two‐way referral systems on hospitalization metrics. In a study involving patients with coronary artery disease within a medical consortium, the experimental group had an average hospitalization duration of 8.18 days. In contrast, the control group had an average hospitalization duration of 9.48 days. The average examination cost was 2101.33 yuan in the experimental group, which was lower than that in the control group (2593.05 yuan). The total hospitalization expenses averaged 53016.80 yuan, which was also lower than those of the control group (55546.20 yuan), and treatment waiting times were significantly reduced [32]. Another study in Shanxi, China, examined patients undergoing surgical treatment for digestive tract cancers. Notably, differences in hospitalization duration and costs were observed among the three groups: direct admission, local physician referral, and expert outreach referral, with hospitalization times of 28.3, 24.2, and 19.2 days, respectively, and costs of 35087.87 yuan, 32853.38 yuan, and 29794.56 yuan [33]. Respectively, some studies employing propensity score matching have revealed that bidirectional referrals do not effectively reduce high‐cost healthcare expenditures, the average annual number of hospitalization days, or out‐of‐pocket hospitalization costs [34]. An analysis of bidirectional referrals in a tertiary care hospital revealed significant differences between upwardly transferred and nontransferred patients in terms of hospitalization days, surgery level, case difficulty, and complications [35]. The transferred patients had an average hospitalization duration of 16.23 days, which was significantly longer than the 11.76 days for nontransferred patients. The proportion of Grade III and IV surgeries was greater among transferred patients (96.58%), indicating more complex and challenging treatments. Difficult cases constituted 34.21% of the transferred patients, which was markedly greater than the percentage of nontransferred patients. Additionally, 37.28% of transferred patients experienced complications, with an average cost of 14641.7 yuan, significantly exceeding that of non‐transferred patients. The discrepancies among the studies may stem from variations in geographic regions, medical association models, sample populations, and research methodologies. Nonetheless, there is a notable scarcity of research on indicators related to the utility benefits of two‐way referrals, which remains a crucial area for future exploration.

### 3.2 Disease Services and Quality Outcomes

A previous study revealed that [20], after adjusting for various patient‐ and hospital‐level confounders, patients with lung, gastric, and esophageal cancers admitted to hospitals within a medical alliance experienced significantly lower risks of adverse outcomes and higher survival probabilities than nonalliance hospitals did. Additionally, another study demonstrated that both loosely and tightly integrated healthcare alliances significantly enhanced the quality of care for patients with hypertension and diabetes. This improvement was particularly pronounced in tightly integrated alliances, where superior clinical skills contributed to better chronic disease management [36]. Research indicates that treatment centers integrated with public hospitals are perceived as offering superior quality compared with those linked with private hospitals [37]. Notably, the quality of care in centers operating under a loosely collaborative model does not significantly differ from that in centers led by public hospitals. It surpasses that of centers associated with private hospitals. Furthermore, a study on hierarchical referral services for hospice care demonstrated that the establishment of hospice agency standards, patient access criteria across all care levels, and evaluation metrics has ensured high‐quality hospice services in primary care, enhanced the quality of patient deaths, and contributed to a more rational allocation of hospice medical resources [38].

### 3.3 Other Aspects of Implementation Effectiveness

A study in Xinjiang demonstrated that [39] telemedicine‐based two‐way referral services for patients with severe coronary artery disease significantly reduced referral times, cutting the average bed wait to approximately 2 h and the total referral time to approximately 14 h. This approach also lowered diagnostic costs, increased patient satisfaction, and decreased overall referral and bed wait times. In Guilin, Guangxi [40], research incorporating two‐way referral indicators such as CMI values and surgical ratios, alongside single‐ and multigroup interrupted time series analysis, has quantitatively evaluated the impact of DRG policy on hierarchical diagnosis and treatment in public hospitals. These findings provide empirical support for improving hierarchical diagnosis and treatment systems. However, the DRG policy remains inapplicable to outpatient graded diagnosis and treatment two‐way referrals.

The effectiveness of two‐way referrals in medical consortia is garnering significant academic interest and has been examined from various perspectives. Nonetheless, quantitative analyses and empirical studies remain limited. The extent of its impact and the factors influencing it require further in‐depth exploration.

## 4 Evaluation of Two‐Way Referrals of Medical Consortia

In evaluating the two‐way referral of medical consortia, a series of specific indicators must be utilized to demonstrate their effectiveness and quality from multiple dimensions. These dimensions encompass the efficiency of referral and the rational allocation of medical resources. The evaluation process should encompass both objective indicators and subjective perspectives. The subjective perspectives should include the viewpoints of both the medical staff providing the services and those of the patients receiving them. This comprehensive approach ensures a multifaceted assessment that can inform improvements in the effectiveness and quality of the two‐way referral process. This multifaceted, multilevel evaluation system facilitates a more comprehensive understanding of and enhancement to the service quality and efficiency of the two‐way referral of medical consortiums, in addition to identifying existing problems.

### 4.1 Indicator Evaluation Tools

From the perspective of the current research on the evaluation index of the effect of two‐way referrals of medical associations, there are more domestic studies in China and fewer foreign studies. A comprehensive literature search was conducted across three major Chinese databases (CNKI, Wanfang, and CQVIP) from January 2019 to December 2023, using MeSH terms including “medical consortium,” “two‐way referral,” and “health policy evaluation.” After applying the inclusion‐exclusion criteria, 10 peer‐reviewed studies were retained for thematic synthesis [41, 42, 43, 44, 45, 46, 47, 48, 49, 50]. Numerous scholars have assessed healthcare consortia through various lenses and methodologies, with two‐way referral indices serving as crucial metrics for evaluating consortium effectiveness. Indicators are developed based on theoretical frameworks such as the “structure‐process‐result” model, stakeholder theory, and the balanced scorecard. Comprehensive evaluations employ methods such as Delphi expert consultation, hierarchical analysis, TOPSIS, RSR, and fuzzy combinations of these techniques, highlighting the diversity in indicator system construction. The evaluation of two‐way referrals, a key aspect of effectiveness, encompasses multiple dimensions, including referral quantity and efficiency, referral quality, patient satisfaction, and collaboration within medical associations. By monitoring and evaluating indicators, issues in the two‐way referral process can be identified, enabling targeted improvements to optimize the medical consortium's operations and enhance referral efficiency and quality. However, current referral indicators lack a focus on quality and efficiency research, necessitating ongoing attention for further in‐depth study (Table 3).

**TABLE 3:** Literature on indicators for evaluating two‐way referrals to medical clusters in China in recent years.

No.	Title	Author	Year	Journal Name	Type of medical association	Dimension	weighted (Y/N)	Specific evaluation indicators related to two‐way referral
1	Evaluation of the implementation effect of hierarchical healthcare of city vertical medical consortium with the application of weighted TOPSIS method	Wu Mian	2024	Medical Education Management	City Medical Association	2	Y	Upward referral process of primary care institutions, downward referral process of core hospitals, green channel (inpatient, outpatient) between referral institutions, special personnel in charge between referral institutions, feedback mechanism between referral institutions, number of upward referrals in accordance with referral criteria, number of downward referrals in accordance with referral criteria, number of referrals from primary care institutions to core hospitals and then back to the primary care institutions, patients' satisfaction with referrals, and the length of waiting time of the primary care clinics' referral bookings Waiting time, referrals for upward and downward referrals, etc.
2	Construction of the Evaluation Index System for Integration of Medical Treatment and Care Services Based on Regional Medical Consortium	LIN Taoyu	2022	Medicine and Society	Medical and Nutritional Care Consortium	3	Y	Establishment of green channels for the elderly within the medical association, establishment of unified two‐way referral standards within the medical association, percentage of two‐way referrals for the elderly within the medical association
3	Construction of Evaluation Index System for Sustainable Development of Medical Alliance–Based on Delphi Method	XIONG Ji‐xia	2021	Health Economics Research	City Medical Association	3	Y	Number and percentage of upward referrals from the primary level, number and percentage of downward referrals from higher‐level hospitals, smoothness of the referral pathway
4	Study on Effect Evaluation of Medical Alliance Reform in a Province	YAO Fang	2021	Health Economics Research	Regional Medical Association	3	Y	The primary referral rate is controlled within a reasonable range, the annual growth rate of the number of referrals from primary medical institutions in the region is more than 10%, lower‐level medical institutions carry out health education work guidance, etc.
5	Evaluation of Primary Medical Service Capacity under the Compact Medical Association Model: a Case Study of Dapeng New District Medical Health Group	ZHU Bin	2023	Chinese Health Quality Management	Regional medical association	3	Y	The number of patients transferred at the district‐municipal level The number of patients transferred at the municipal and district levels Social well‐being—the number of patients transferred at the district level District‐level—number of patients who have been transferred to community health
6	Evaluation on the operation of loose medial alliance based on DRGs	ZHANG Guo‐hua	2020	Soft Science of Health	Loose medical association	2	N	Number of upward transfers, number of downward transfers
7	Research on the construction of the hierarchical medical system and treatment performance evaluation model based on grounded theory and structural equation	LUO Da	2023	Chinese Hospitals	Regional medical association	3	N	Referral of patients from higher‐level hospitals to lower‐level hospitals, referral of patients from primary‐level hospitals to higher‐level hospitals, degree of co‐operation between higher and lower‐level hospitals, direct referral rate, degree of co‐operation with referrals, degree of satisfaction with referrals, etc.
8	Performance Evaluation Indicators Screening for the Vertical and Compact Medical Consortium	WANG Manli	2020	Chinese General Practice	Compact medical association	2	N	Proportion of patients with booked referrals to the total number of referrals, proportion of referrals enjoying priority hospitalization to the total number of referrals for inpatient admission, proportion of downward referrals from higher level hospitals to their total inpatient admissions, proportion of upward referrals from primary healthcare institutions to the number of consultations and treatments at core hospitals
9	Research of Performance Evaluation of Urban and County Medical Alliance in China	LIU Chun‐ping	2021	Chinese Hospital Management	City and County Healthcare Consortiums	2	Y	Number of patients referred from member hospitals to lead hospitals; number of patients referred from lead hospitals to member hospitals
10	Evaluation of Hierarchical Diagnosis and Treatment Effect and Influencing Factors of Beijing Medical Alliance	Hu Tong	2024	Chinese Hospital Management	City Medical Association	3	Y	Two‐way referral mechanism, number of two‐way referrals, quality and effectiveness of two‐way referrals, whether there is an upward referral process in primary care institutions, whether there is a downward referral process in core hospitals, whether there is a green channel (inpatient and outpatient) between referral institutions, whether there is a special person in charge of the referral process between referral organizations, whether there is an information feedback mechanism between referral organizations, number of referrals that meet the referral criteria, the number of referrals that meet the referral criteria, number of referrals from primary level to core hospitals and back to primary level, patient satisfaction with the referral, waiting time for referral appointments for primary care outpatients

### 4.2 Evaluation of Medical Staff

Regional disparities exist in the knowledge and satisfaction levels of medical personnel regarding the two‐way referral system. In Qingpu District, Shanghai, 89.54% of the doctors were familiar with the system's standards and processes, yet only 57.25% expressed satisfaction with it [51]. Another study in Shanghai reported that only 20.8% of physicians were willing to accept downward referrals, which was influenced by factors such as education level, understanding of the system, and perceptions of hospital resources [52]. In Ningbo city, Zhejiang Province, 79.1% of medical staff were knowledgeable about two‐way referrals, but only 45.3% were satisfied [27]. At the First Affiliated Hospital of Chongqing, 93.2% of medical staff understood the medical consortium and two‐way referral system, with 53.5% having experience in providing referral services, resulting in 151 cases of two‐way referrals [53]. In a survey of 1280 medical staff from various hospitals across 21 prefectures and municipalities in Sichuan Province, 869 (68.9%) did not engage in medical consortium services [54]. In Shenzhen, Zhanjiang, and Dongguan in Guangdong Province, approximately 39.9% of doctors employed by hospitals and community health organizations within specific healthcare consortia had no prior experience with downward referrals. A total of 15.4% of the doctors in community health organizations had not encountered upward referrals [55]. A study in Anhui involving 511 medical staff from core hospitals in three urban medical clusters revealed that only 139 (27.2%) were satisfied with the current two‐way referral system, indicating low satisfaction levels [56]. In Anyang city, Henan Province, there remains significant room for improvement in the perception and implementation of two‐way referrals, particularly as higher‐ranking medical staff show decreased willingness for downward referrals [57].

Several studies and analyses have identified key factors influencing the two‐way referral of medical staff within medical clusters [15, 56, 58, 59, 60]: policy awareness and promotion, inadequate grassroots service capacity, unclear referral criteria, economic benefit disparities, patient trust and willingness, and job and income satisfaction among medical staff. Overall, the operational effectiveness of two‐way referrals has yet to meet expectations. To increase medical staff satisfaction and engagement in the referral system, it is essential to not only bolster policy awareness and training but also optimize referral criteria, improve grassroots service capacity, balance economic benefits, and increase the working conditions and income of medical staff. These efforts are crucial for the effective implementation of the two‐way referral system.

### 4.3 Patient Evaluation

A previous study [61] reported that community health workers presented the highest level of awareness of the two‐way referral policy, with 138 individuals (86.79%) being knowledgeable about it. In contrast, patients demonstrated the lowest level of awareness, with only 81 individuals (27.92%) being informed and 32.07% being utterly unaware of the policy. In a survey of patients in Ningbo, Zhejiang Province [27], the majority of patients, 78.8%, surpassed the “understanding” level regarding the medical association; yet, 60% had not been referred to it. Another study revealed that 80.33% (388/483) of residents had never visited a community hospital, highlighting a lack of awareness and understanding of the first‐visit model within the healthcare consortium [62]. In Shanghai, only 37.6% (326/866) of patients were open to downward referrals, which was influenced by economic factors, marital status, and recognition of the community‐based priority treatment system [52]. Among the 547 residents with referral experience [18], 474 (86.7%) had upward referrals, whereas 106 (19.4%) had downward referrals, indicating a predominant “upward” referral trend. The existing referrals present a unidirectional referral situation in which “it is easy to go up but difficult to go down.” A study conducted in Shandong Province on the propensity of elderly individuals to engage in downward referrals and the influencing factors revealed that out of 1198 participants, only 28.7% initiated downward referrals, whereas 33.9% expressed readiness to accept such referrals based on medical advice [63]. Similarly, research on patient self‐referral patterns within the rural healthcare system of Qianjiang, Chongqing, demonstrated that fostering a lasting rapport with a designated primary care provider can cultivate trust between patients and healthcare professionals, consequently curbing the unnecessary utilization of healthcare resources [64]. Moreover, findings from a separate investigation revealed a staggering 78.9% rate of redundant examinations in community‐based general hospitals [65]. Overall, the study highlighted various determinants influencing patients' bidirectional referral behavior, encompassing medical practices, trust inclinations, financial considerations, holistic healthcare metrics, social welfare healthcare structures, inadequate awareness of referral protocols and criteria, intricate procedures, and restricted autonomy in selecting referral hospitals [66, 67].

## 5 Summary and Outlook

The hierarchical diagnosis and treatment system is a recognized and efficient medical service model globally. Although China adopted this system relatively late, the national focus and rapid development have accelerated the establishment of integrated healthcare networks, optimized resource allocation, and enhanced service efficiency. In particular, the service capabilities of primary healthcare facilities have significantly improved [68]. Nevertheless, the two‐way referral system within healthcare networks, as part of the hierarchical diagnosis and treatment framework, encounters several challenges. The volume of two‐way referrals in China remains relatively low. First, China's bilateral referral volume remains relatively low, highlighting notable disparities in the effectiveness of various medical clusters across regions. The absence of standardized criteria and norms has hindered the establishment of a uniform referral pathway and a dynamic adjustment mechanism. Second, the current evaluation system has structural deficiencies because it emphasizes referral quantity over quality; notably, it lacks a case‐mix index (CMI) assessment grounded in diagnosis‐related groups (DRGs). This deficiency has led to skewed benefit analyses, diminishing evaluation accuracy and practicality. Third, research perspectives are constrained temporally and spatially, predominantly focusing on short‐term or localized effects while neglecting long‐term and global impacts; thus, continuous monitoring is lacking. Finally, insufficient incentives for key stakeholders' engagement persist, with patients slowly adopting new medical care paradigms due to low trust in primary healthcare facilities, resulting in diminished motivation, satisfaction, and inadequate recognition of medical personnel. Furthermore, performance evaluations, medical insurance policies, government directives, and obligatory regulations often fail to adequately constrain healthcare institutions at all levels and patient referrals.

During the crucial timeframe of the “14th Five‐Year Plan” for health implementation, which coincides with the pivotal phase for establishing China's hierarchical diagnosis and treatment system, it is imperative to consider the following dimensions to devise an innovative approach to address the challenges associated with the two‐way referral system. First, enhancing the foundation of primary healthcare services is crucial, emphasizing the development of human resources through intensified training for general practitioners, enhancing the competency of primary medical staff, improving salaries and benefits, and establishing professional titles. The primary focus should be on enhancing training programs for general practitioners to increase their skills, compensation, benefits, and professional recognition. Second, enhancing resource sharing and collaboration is essential and requires clear delineation and reinforcement of the roles of hospitals at all levels to establish a comprehensive health management system throughout patients' life cycles. This system should prioritize healthcare services, medical technology, and population health, leveraging the strengths of healthcare institutions at all levels. Furthermore, health insurance payments and pricing reforms can be advanced by adjusting DRGs/DIP payment coefficients, evaluating the compliance rate of two‐way referrals, and utilizing incentives to guide the rational allocation of medical resources and patient flow. Finally, a comprehensive evaluation system that includes clinical outcome indicators, quality and safety indicators, cost‐effectiveness indicators, and patient‐reported outcomes should be implemented. Advanced technologies, such as big data, blockchain, and artificial intelligence, are utilized to ensure the credibility of data traceability.

In conclusion, within the tiered diagnosis and treatment system framework, addressing the challenges of the two‐way referral system requires bolstering the primary medical service infrastructure, fostering resource sharing and collaboration, advancing reforms to the health insurance contribution mechanism, and establishing a comprehensive evaluation system to facilitate the appropriate allocation of medical resources and patients. This endeavor has profound implications for enhancing the efficiency and equity of the healthcare system, which are pivotal in improving healthcare service quality and effectiveness in China. Moreover, this research offers valuable insights for other nations and territories seeking to implement hierarchical diagnosis and treatment systems and refine referral procedures.

## Conflicts of Interest

The authors declare no conflicts of interest.
